# Site-selective ^13^C labeling of histidine and tryptophan using ribose

**DOI:** 10.1007/s10858-017-0130-9

**Published:** 2017-08-30

**Authors:** Ulrich Weininger

**Affiliations:** 1 Department of Biophysical Chemistry, Center for Molecular Protein Science, Lund University, P. O. Box 124, 22100 Lund, Sweden; 2 Institute of Physics, Biophysics, Martin-Luther-University Halle-Wittenberg, 06120 Halle (Saale), Germany

**Keywords:** NMR, Relaxation, Protein dynamics, Aromatic side chain, Isotope labeling

## Abstract

Experimental studies on protein dynamics at atomic resolution by NMR-spectroscopy in solution require isolated ^1^H-X spin pairs. This is the default scenario in standard ^1^H-^15^N backbone experiments. Side chain dynamic experiments, which allow to study specific local processes like proton-transfer, or tautomerization, require isolated ^1^H-^13^C sites which must be produced by site-selective ^13^C labeling. In the most general way this is achieved by using site-selectively ^13^C-enriched glucose as the carbon source in bacterial expression systems. Here we systematically investigate the use of site-selectively ^13^C-enriched ribose as a suitable precursor for ^13^C labeled histidines and tryptophans. The ^13^C incorporation in nearly all sites of all 20 amino acids was quantified and compared to glucose based labeling. In general the ribose approach results in more selective labeling. 1-^13^C ribose exclusively labels His δ2 and Trp δ1 in aromatic side chains and helps to resolve possible overlap problems. The incorporation yield is however only 37% in total and 72% compared to yields of 2-^13^C glucose. A combined approach of 1-^13^C ribose and 2-^13^C glucose maximizes ^13^C incorporation to 75% in total and 150% compared to 2-^13^C glucose only. Further histidine positions β, α and CO become significantly labeled at around 50% in total by 3-, 4- or 5-^13^C ribose. Interestingly backbone CO of Gly, Ala, Cys, Ser, Val, Phe and Tyr are labeled at 40–50% in total with 3-^13^C ribose, compared to 5% and below for 1-^13^C and 2-^13^C glucose. Using ribose instead of glucose as a source for site-selective ^13^C labeling enables a very selective labeling of certain positions and thereby expanding the toolbox for customized isotope labeling of amino-acids.

**Electronic supplementary material:**

The online version of this article (doi:10.1007/s10858-017-0130-9) contains supplementary material, which is available to authorized users.

## Introduction

NMR spectroscopy enables high resolution studies of protein structures (Wuthrich 2001), dynamics (Palmer 2004) and interactions (Zuiderweg 2002). A key requirement for studies of protein dynamics, that are often directly linked to function (Mittermaier and Kay 2006), are isolated ^1^H-X spin pairs that are not affected by coupling with their neighbours. While being the default for dynamic studies of backbone amides (Akke and Palmer 1996; Ishima and Torchia 2003; Jarymowycz and Stone 2006; Loria et al. 1999), dynamics studies of amino acid side chains (Hansen and Kay 2011; Hansen et al. 2012; Lundstrom et al. 2009a; Millet et al. 2002; Muhandiram et al. 1995; Mulder et al. 2002; Paquin et al. 2008; Weininger et al. 2012a, c) often requires site selective ^13^C and/or ^2^H labeling (Lundstrom et al. 2012b). Studies of side chain dynamics not only complement existing backbone studies, but widen the view on certain processes and enable unique additional information of structure (Korzhnev et al. 2010; Neudecker et al. 2012), ring-flips (Weininger et al. 2014b; Yang et al. 2015), histidine tautomers (Weininger et al. 2017) and proton occupancy and transfer reactions (Hansen and Kay 2014; Wallerstein et al. 2015). For studies of structure, interaction and function site selective labeling is not strictly required but often advantageous, especially for large systems (Lundstrom et al. 2012a; Ruschak and Kay 2010; Tugarinov and Kay 2005) or in solid -state (Eddy et al. 2013).

In the most general way site-selective ^13^C labeling is achieved using glucose (Lundstrom et al. 2007; Teilum et al. 2006), glycerol (Ahlner et al. 2015), or pyruvate (Milbradt et al. 2015). These labeling schemes with precursors at the beginning of the biological pathways in bacteria, label many positions in all amino acids. Using precursors closer to the desired product result in a more exclusive labeling of certain positions. A well established case is the exclusive site selective labeling of methyl groups at high yields which results in superb NMR probes (Ruschak and Kay 2010; Tugarinov et al. 2006; Tugarinov and Kay 2005; Weininger et al. 2012b). Aromatic side chains can be targeted specifically by erythrose labeling (Kasinath et al. 2013; Weininger 2017) and more advanced chemically synthesized precursors for labeling of Trp (Schörghuber et al. 2015), Tyr and Phe (Lichtenecker et al. 2013) and most recently for His (Schörghuber et al. 2017). Also advanced in-vitro strategies using the SAIL approach have been developed for Trp (Miyanoiri et al. 2011), Tyr and Phe (Takeda et al. 2010).

Aromatic residues are an interesting target. They are bulky and form a substantial part of protein hydrophobic cores. They are also over-represented in binding sites (Lo Conte et al. 1999). Especially Tyr and Trp contribute significantly to the binding free energy (Bogan and Thorn 1998). They can be involved in specific aromatic–aromatic pair interactions (Burley and Petsko 1985, 1989), forming hydrogen bonds (Levitt and Perutz 1988), or interacting with cations (Mahadevi and Sastry 2013) or sulfur atoms (Valley et al. 2012). His and Tyr play important catalytic residues for enzyme activity (Bartlett et al. 2002). His has a p*K*_a_ value close to physiological pH and can exist in three different states, one protonated and two different tautomeric neutral forms (Reynolds et al. 1973). It can act as a nucleophile, an acid/base catalyst (Fersht 1977), as a proton shuttle (Lindskog 1997), and a an hydrogen bond donor and acceptor (Krishna Deepak and Sankararamakrishnan 2016; Preimesberger et al. 2015).

Recently improved NMR methods ^13^C based aromatic side chain dynamics have been developed (Weininger et al. 2012a). The first studies of order parameters have been reported (Boyer and Lee 2008; Kasinath et al. 2013, 2015) and experiments to characterize dynamics on the ms (Weininger et al. 2012c) and µs (Weininger et al. 2014a) time-scales have been developed. Also site selective labeling has improved their use as structural probes (Milbradt et al. 2015) and residual dipolar couplings in aromatic side chains have been measured (Sathyamoorthy et al. 2013).

Here we present an easy and robust alternative approach using selectively labeled ribose in combination with unlabeled glucose. This approach is very close to standard ^13^C labeling using glucose. The only modification is the additional presence of ribose. Further, we quantify the ^13^C incorporation in all positions of the 20 amino acids. 1-^13^C ribose labeling leads to an exclusive labeling of Trp δ1 and His δ2 in aromatic side chains. His δ2 is an excellent probe for the tautomeric state of an histidine (Pelton et al. 1993; Vila et al. 2011; Weininger et al. 2017) Further these are the only positions in aromatic side chains that are per default immune against strong ^1^H-^1^H coupling artifacts in relaxation dispersion experiments (Weininger et al. 2013). The incorporation yield (37%) is however lower compared to 2-^13^C glucose (50%). Histidine positions β, α and CO become significantly labeled at around 50% in total by 3-, 4- or 5-^13^C ribose. His β does not become labeled at all using well established 1-^13^C or 2-^13^C glucose protocols and only 60% of this yield using 2-^13^C erythrose. Using ribose His Cβ becomes accessible for dynamics on the ms time-scale (Lundstrom et al. 2009b). Interestingly backbone CO of Gly, Ala, Cys, Ser, Val, Phe and Tyr are labeled at 40–50% in total with 3-^13^C ribose, compared to 5% and below for glucose. Also ribose seems to enter the chorismate pathway.

Finally, we show that the ribose-based approach for site-selective ^13^C labeling can be easily combined with the glucose approach, enabling a more custom labeling. A combined 1-^13^C ribose and 2-^13^C glucose labeling yields a isolated ^13^C incorporation in His δ2 of 75%.

## Materials and methods

### Expression and purification

Recombinant FKBP12 was expressed and purified as described (Weininger 2017). M9 minimal medium was subsidized at the beginning with 1 g/l ^15^N NH_4_Cl, 2 g/l unlabeled glucose 2 g/l selectively ^13^C enriched ribose, unless otherwise indicated. At the end the buffer was exchanged to NMR buffer and the protein was concentrated to ~12 mg/ml.

### NMR spectroscopy

All spectra were run on 900 µM samples in 25 mM sodium phosphate, pH 7.0 and 10% (v/v) D_2_O at 25 °C and a static magnetic field strength of 14.1 T. For each sample, a ^1^H–^15^N plane of an HNCO, non-ct ^1^H–^13^C HSQCs for the aliphatic and aromatic regions, and a 1D spectrum on ^13^C were recorded for quantification of ^13^C incorporation. Intensities of different samples were referenced to intensities of a ^1^H–^15^N HSQC to account for small concentration deviations in the samples. Aromatic ^13^C relaxation studies were performed using L-optimized TROSY detected relaxation experiments (Weininger et al. 2012a). All spectra were processed using NMRPipe (Delaglio et al. 1995) and analysed using NMRView (Johnson 2004).

### Data analysis

^13^C incorporation was resulting from ribose labeling was compared to glucose labeling (Weininger 2017). All positions of interest described in this article resulting from ribose labeling (and glucose labeling for comparison) were isolated and showed no signs of any ^13^C–^13^C ^1^J coupling. Intensities were normalized to the fully ^13^C enriched sample and expressed in %. By analysing multiple signals of the same kind, the relative error in the intensities of ^13^C covalently bound to ^1^H could be estimated to 1%. Errors for ^13^C not bound to ^1^H were estimated to 3%.

## Results and discussion

**Fig. 1:**
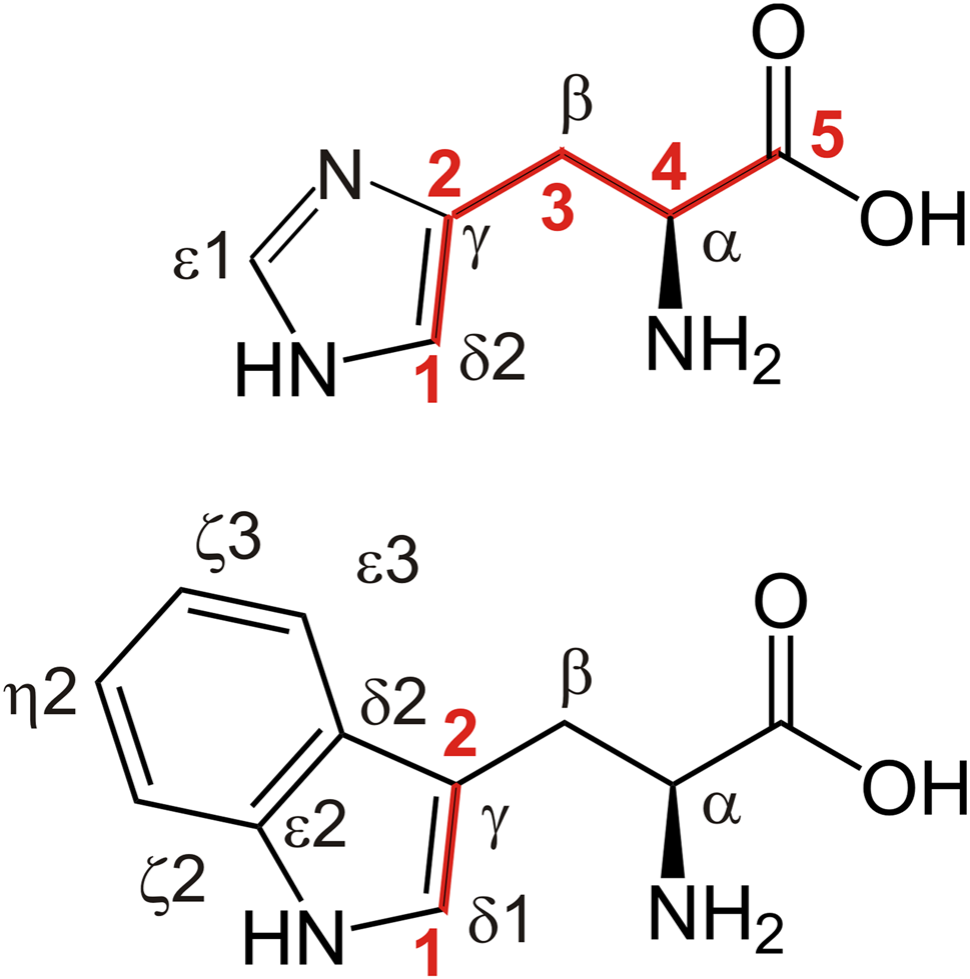
Site-selective ^13^C incorporation using site-selectively labeled ribose. Histidine and tryptophan are shown with the positions labeled. Incorporation of carbons from ribose is shown in *red*, with the positions of ribose (1–5) labeled

Ribose is a precursor that directly enters the pentose-5-phosphate way from which histidine and parts of tryptophan are built (Fig. 1 and SI Fig. 1 for more detail). This allows for a very distinct labeling of only the positions of interest. To make the labeling procedure as general and simple as possible and to avoid scrambling from ribose to other pathways, selective ^13^C labeled ribose is used in combination with unlabeled glucose. Further this allows for a possible combination of selective ^13^C ribose and glucose based labeling in a straightforward way. ^13^C incorporation was monitored for all side-chain positions, with exception of Tyr γ, His γ, and Trp δ2 and ε2. They all lack a directly attached proton which makes them harder to study and therefore less interesting. The resulting data provides information on background labeling, scrambling, and unexpected selective incorporations, as described below.

### Site-selective ^13^C labeling of histidine and tryptophan

**Fig. 2:**
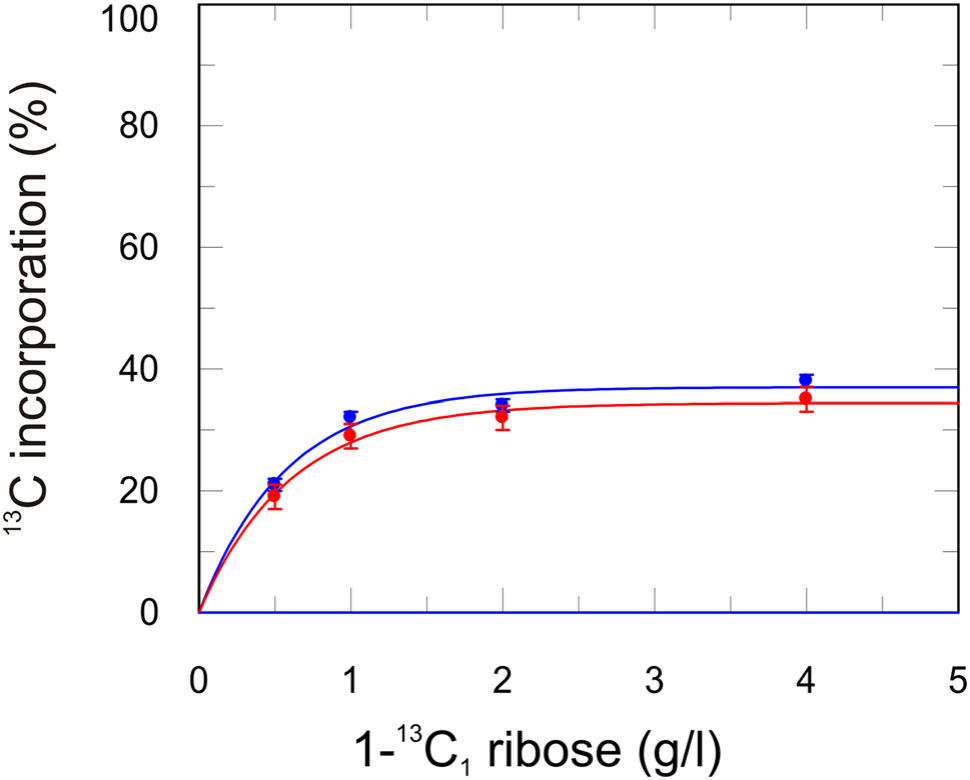
^13^C incorporation level in aromatic side-chains resulting from different amounts of 1-^13^C ribose in the medium. Incorporation His δ2 (*blue*) and Trp δ1 (*red*) are shown in % relative to fully ^13^C enriched glucose. *Solid lines* are single exponential fits

The above mentioned ribose labeling strategy leads to following isolated ^13^C labeling at the expected positions (Fig. 1) and the background labeling of other positions is much less than that obtained using glucose as the sole carbon source. The optimal amount of labeled ribose in the expression medium was tested using different amounts of 1-^13^C_1_-ribose (Fig. 2). A virtual maximum in ^13^C incorporation is at 2 g ribose per liter medium, whereas already at 1 g/l one is close to the maximum. 1 g/l seems to be the most economic concentration for close to optimal ^13^C incorporation per ribose needed. However one can still slightly increase the level of ^13^C incorporation by adding more ribose. In this study all (^13^C-site labeling) quantifications are done with 2 g/l ribose.

**Fig. 3:**
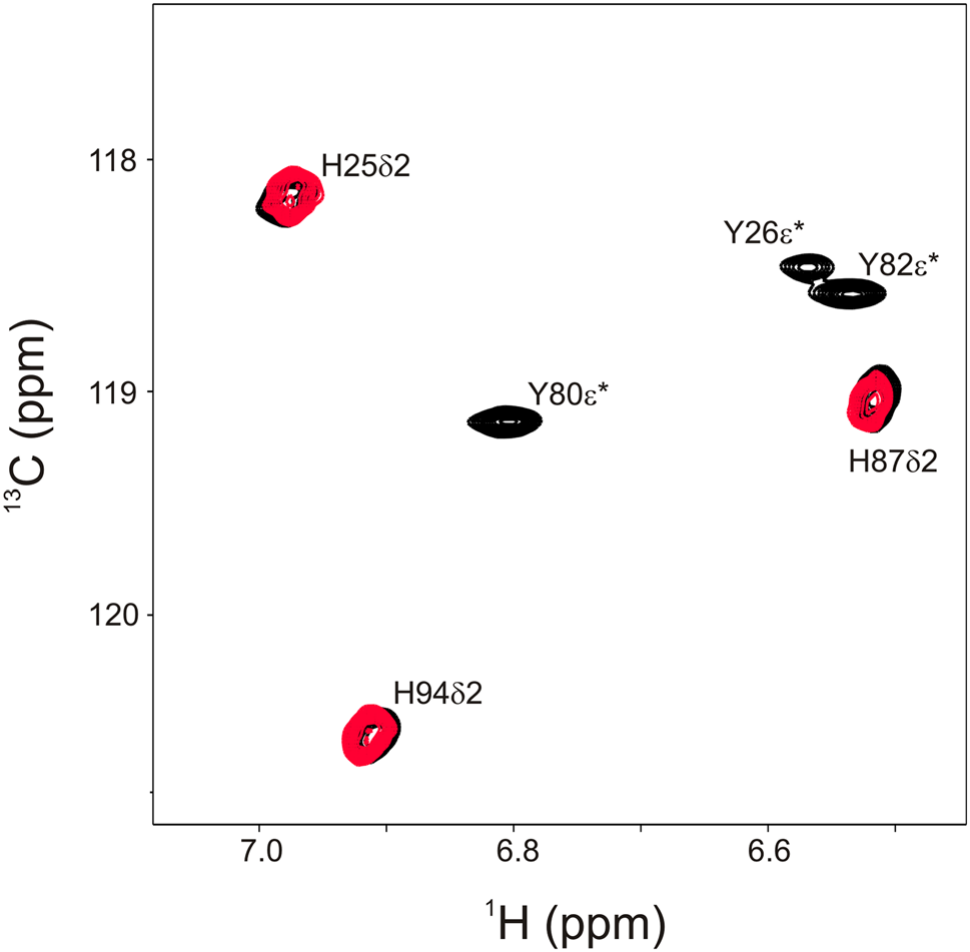
Tyr ε* His δ2 region of an aromatic ^1^H^13^C-HSQC of FKBP12. Signals arising from a 2-^13^C_1_-glucose labeled sample are shown in *black*, while signals from a 1-^13^C_1_-ribose labeled sample are shown in *red*. His δ2 signals are broadened because ^15^N was not decoupled. *Asterisk* represents an averaged signal of position 1 and 2 because of fast exchange of the aromatic rings on the NMR time-scale

^13^C incorporation levels for the expected positions in His and Trp (see Fig. 1) are summarized in Table 1 (incorporation levels for all positions and amino acids using ribose labeling are listed in SI Table). For His δ2 and Trp δ1 the ^13^C incorporation using 1-^13^C ribose are 38 and 35%, respectively. This is a clear improvement compared to 1-^13^C glucose (26 and 26%), but doesn’t reach the yield of 2-^13^C glucose (52 and 49%). 2-^13^C glucose also results in isolated ^13^C positions which wasn’t clear from previous studies (Lundstrom et al. 2007). One potential problem of 2-^13^C glucose is, that it is effectively labeling Tyr ε* as well, which resonate in the same region as His δ2. 1-^13^C ribose however labels His δ2 exclusively (Fig. 3). Both His δ2 and Trp δ1 are not affected by ^1^H-^1^H strong coupling artifacts in relaxation dispersion experiments (Weininger et al. 2013) and His δ2 is a powerful probe for tracking the tautomeric state of histidines (Pelton et al. 1993; Vila et al. 2011; Weininger et al. 2017). Additionally ^13^C ribose enriched on positions 2–5 yields to very efficient and isolated labeling of Trp and His γ (though not directly shown for His), His β, His α and His CO. Especially His β is very useful since it doesn’t get isolated ^13^C labeled by 1-^13^C and 2-^13^C glucose and far less by 2-^13^C erythrose. Moreover His β is the only position that gives rise to signal in an aliphatic ^1^H^13^C HSQC that gets labeled above 3%, which means basically natural abundance. His CO seems to be labeled extremely efficient (71%) by 5-^13^C ribose while all other CO are below 15%. This might be a useful feature for selective HNCO experiments.

**Table 1:** Site-selective ^13^C incorporation in histidine and tryptophan using ribose

	1-^13^C	2-^13^C	3-^13^C	4-^13^C	5-^13^C
His CO	1	4	4	5	71
Hisα	3	3	0	42	1
His β	2	3	56	1	1
His γ	n.d	n.d	n.d	n.d	n.d
His δ2	38	7	1	2	1
Trp γ	3	34	0	3	0
Trp δ1	35	6	2	1	2

Values are in %. Errors are estimated to 1% for ^1^H bound ^13^C, 3% for others (Trp γ). 1% for non labeled positions is expected because of natural abundance of ^13^C

### ^13^C relaxation of aromatic side chains

**Fig. 4:**
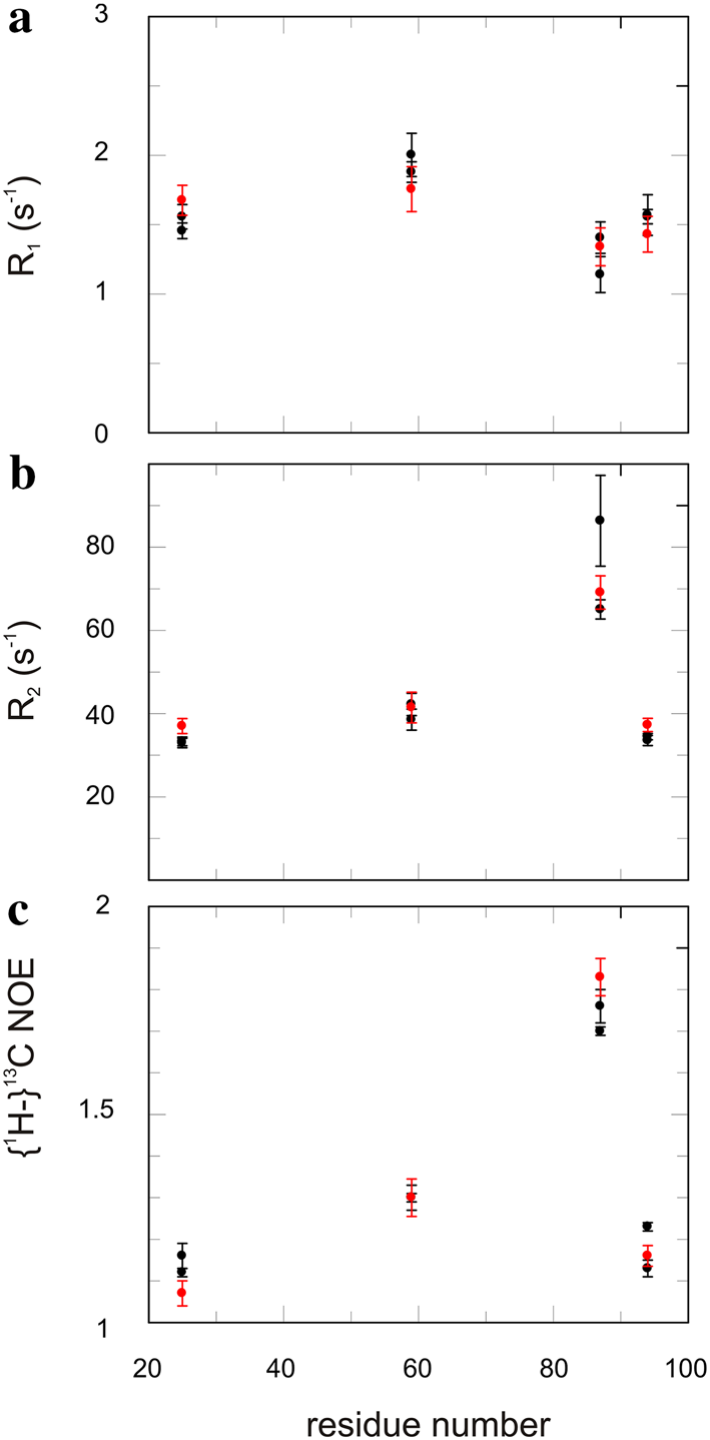
Comparison of aromatic ^13^C relaxation experiment using glucose or ribose labeled FKBP12. *R*_1_ (**a**), *R*_2_ (**b**) and {^1^H-}^13^C NOE (**c**) experiments were conducted using site-selective labeled FKBP12 based on 1-^13^C and 2-^13^C (*black*) glucose or 1-^13^C ribose (*red*) labeling

Both ribose and glucose labeling lead to site-selective ^13^C labeling in aromatic side-chains of Trp and His. By comparing *R*_1_, *R*_2_ and ^13^C NOE (Ferrage et al. 2008) for identical positions between ribose- and glucose-labeled samples, we observe an excellent agreement (Fig. 4). Thus, the two approaches give virtually the same result; potential long range ^13^C-^13^C couplings do not affect the results. While it is not clear if additional deuteration is needed for artifact free relaxation data (Kasinath et al. 2013) or not (Weininger et al. 2012a) in general, this will not affect aromatic positions that get labeled with ribose. Both His δ2 and Trp δ1 do only have one proton in ^2^J distance of the 13C of interest. This protons are nitrogen bound and exchange with the solvent. If they matter one has to change the solvent but not the labeling protocol. ^13^C relaxation dispersion experiments both for CPMG (Weininger et al. 2012c) and *R*_1ρ_ (Weininger et al. 2014a) were previously validated for glucose labeled samples. These experiments can be directly applied to samples resulting from ribose labeling, since the relaxation behaviour is identical.

### Site-selective ^13^C labeling in non standard positions

Since ribose is a precursor closer to the end product then glucose the ^13^C background in other then the desired positions (Fig. 1) is much reduced (SI Table 1). However, a few positions are worth mentioning, which become efficiently labeled with ^13^C. In contrast to glucose all positions labeled with ribose appear to result in isolated ^13^C, no signs of ^13^C-^13^C couplings could be detected. 1-^13^C ribose only labels Tyr ζ above 10%. Since Phe ζ doesn’t show any significant ^13^C incorporation this might be a false positive resulting from a less reliable ^13^C direct detected 1D experiment. 2-^13^C ribose only labels Tyr ε and Phe ε to around 15%, indicating some cross over to the chorismate pathway. Indeed ribose 5-phosphate can be transformed to erythrose 4- phosphate via sedoheptulose 7-phosphate by transketolase transaldolase and transaldolase. (Schwender et al. 2003) 3-^13^C ribose leads to a significant ^13^C incorporation (30–50%) in the backbone carbonyl of Gly, Ala, Cys, Lys, Val, Trp, Phe and Tyr. 4-^13^C and 5-^13^C ribose show some weak incorporation pattern of 2-^13^C and 1-^13^C glucose, respectively. Despite the backbone carbonyl none of the positions show a higher or even close ^13^C incorporation compared to glucose. However they result in spectra with a reduced amount of signals and any ^13^C-^13^C couplings.

### Combined labeling of ribose and glucose

Since the described labeling scheme is based on ^13^C labeled ribose and unlabeled glucose and the scrambling from ribose into other pathways is low, ^13^C labeling both from ribose and glucose can be easily combined. This was demonstrated in an approach where protein was expressed using 2 g/l 1-^13^C ribose and 2 g/l 2-^13^C glucose. Both precursors are labeling aromatic His δ2 and Trp δ1, while 2-^13^C glucose is additionally labeling Trp ζ3 and ζ2 and Phe and Tyr ε*. Theoretical considerations expect a labeling yield in His δ2 and Trp δ1 of about 70%: About 37% of histidine is produced from 1 to 13C ribose with 99% ^13^C incorporation in δ2 and about 63% is produced from 2 to 13C glucose with 51% ^13^C incorporation in δ2. By this approach one would maximizes the ^13^C labeling of His δ2. Of course this is just useful if signals from His δ2 are isolated from Tyr ε*. The experiment confirms this considerations. 75% of His δ2 and Trp δ1 get site selectively ^13^C labeled. This approach is generating samples with the highest sensitivity of isolated His δ2 and Trp δ1, outperforming the 2-^13^C glucose approach by 50% and thus nicely expanding the toolkit for a more customized site selective ^13^C labeling.

### Different ways of site-selective ^13^C labeling of histidine and tryptophan

Up to date there are three different approaches of site-selective ^13^C labeling of histidine (CO, α, β, δ2) and tryptophan (δ1). The most general is 2-^13^C glucose (Lundstrom et al. 2007) which effectively (around 50%) labels His α and δ2, as well as Trp δ1. Additionally different aromatic sites (Phe and Tyr ε, and Trp ζ3 and ζ2) and α positions (all except Leu) get ^13^C labeled and accessible for NMR dynamic studies as well. The other two, using ribose (this work) or precursors closer to the products (Schörghuber et al. 2015, 2017) are more discriminating in the positions that get ^13^C labeled and can thereby solve potential overlap problems.

No precise values of ^13^C incorporation have been reported for the latter approaches (Schörghuber et al. 2015, 2017) nor have all positions been targeted (Trp δ1, and His α, β and δ2 are still missing). However this seems relatively straight forward to achieve and could be superior, because the starting compounds are closer to the products. The ribose approach (this work) has the disadvantage of a lower ^13^C incorporation in His δ2 and Trp δ1 (37%), is about the same for His α, and superior for His β and His CO, compared to the 2-^13^C glucose approach. If wanted ^13^C incorporation in His δ2 and Trp δ1 can be maximized to 75% at the cost of not selectively targeting these position anymore.

The ribose approach is about twice as expensive (for His δ2 and Trp δ1, and more for other positions) as the glucose approach, the compounds by Schörghuber require organic synthesis. Both effect the use as a standard method at the moment, but this should improve if they get more established. Even now they are very useful and superior for certain applications (overlap or sensitivity issues, new positions available). Since these compounds are just added to the regular expression medium, their use is as straight forward as any glucose labeling. They both label aromatic sites highly selective (Trp δ1 and His δ2 for ribose, Trp δ1 or His δ2 for Schörghubers compounds, after some adaptation), however the approach by Schörghuber is more discriminating for His CO.

## Conclusions

We have shown that ribose as a source for site-selective ^13^C labeling of histidine and tryptophan yields more selective incorporation patterns than what is achieved using glucose. By this it is possible to study aromatic His δ2 signals, that are very diagnostic of the tautomeric states of histidine, without possible interference of Tyr ε* signals. If there is no interference one can maximize (75%) the ^13^C incorporation in His δ2 and Trp δ1 by a combination of 1-^13^C ribose and 2-^13^C glucose. Further ribose labeling leads to an improved site selective ^13^C incorporation in the aliphatic moiety of histidine compared to the glucose approach. Especially His β, which is not accessible by the standard 1-^13^C or 2-^13^C glucose approach, becomes significantly ^13^C labeled with 56% and available studies of dynamics.

## Electronic supplementary material

Below is the link to the electronic supplementary material.

Supplementary material 1 (DOCX 3497 KB)
