# Supplementary material for: Site-selective 13C labeling of histidine and tryptophan using ribose
Source: J Biomol NMR. 2017 Aug 30;69(1):23–30. doi: 10.1007/s10858-017-0130-9 (PMC5626788; doi:10.1007/s10858-017-0130-9)
Supplement: Supplementary file 1 — Supplementary material 1 (DOCX 3497 KB) [file 10858_2017_130_MOESM1_ESM.docx]

**Site-selective ^13^C labeling of Histidine and Tryptophan Using Ribose**

**Ulrich Weininger**^1,2^

^1^Department of Biophysical Chemistry, Center for Molecular Protein Science, Lund University, P.O. Box 124, SE-22100 Lund, Sweden

^2^Institute of Physics, Biophysics, Martin-Luther-University Halle-Wittenberg, D-06120 Halle (Saale), Germany

e-mail: ulrich.weininger@physik.uni-halle.de

phone: +49 345 55 28555

fax: +49 345 55 27161





**SI Fig 1**: Excerpt of the biosynthetic pathways from ribose to histidine and tryptophan. Positions arising from ribose are labeled in red.

**SI Table 1**: Site-selective ^13^C incorporation using ribose.

| position | 1-^13^C | 2-^13^C | 3-^13^C | 4-^13^C | 5-^13^C |
| --- | --- | --- | --- | --- | --- |
| Phe CO | 0 | 9 | 51 | 4 | 4 |
| Phe α | 6 | 5 | 2 | 18 | 2 |
| Phe β | 4 | 2 | 1 | 1 | 17 |
| Phe γ | -1 | 8 | 0 | 16 | 1 |
| Phe δ* | 4 | 2 | 1 | 1 | 25 |
| Phe ε* | 5 | 15 | 2 | 18 | 1 |
| Phe ζ | 0 | 1 | 27 | 1 | 1 |
|  |  |  |  |  |  |
| Tyr CO | 1 | 8 | 50 | 5 | 4 |
| Tyr α | 0 | 5 | 0 | 22 | 1 |
| Tyr β | 3 | 2 | 1 | 1 | 21 |
| Tyr γ |  |  |  |  |  |
| Tyr δ* | 5 | 2 | 1 | 1 | 28 |
| Tyr ε* | 5 | 14 | 1 | 13 | 1 |
| Tyr ζ | 15 | 6 | 36 | 9 | 4 |
|  |  |  |  |  |  |
| Trp CO | 1 | 10 | 44 | 7 | 5 |
| Trp α | 2 | 4 | 1 | 19 | 2 |
| Trp β | 6 | 2 | 2 | 3 | 23 |
| Trp γ | 3 | 34 | 0 | 3 | -1 |
| Trp δ1 | 35 | 6 | 2 | 1 | 2 |
| Trp δ2 |  |  |  |  |  |
| Trp ε2 |  |  |  |  |  |
| Trp ε3 | 6 | 2 | 1 | 1 | 23 |
| Trp ζ3 | 3 | 5 | 1 | 24 | 2 |
| Trp η2 | 5 | 20 | 2 | 1 | 1 |
| Trp ζ2 | 3 | 1 | 16 | 1 | 2 |
|  |  |  |  |  |  |
| His CO | 1 | 5 | 5 | 5 | 71 |
| His α | 3 | 3 | 0 | 42 | 1 |
| His β | 2 | 3 | 56 | 1 | 1 |
| His γ |  |  |  |  |  |
| His δ2 | 38 | 7 | 1 | 2 | 1 |
| His ε1 | 3 | 1 | 1 | 2 | 8 |
|  |  |  |  |  |  |
| Gly CO | 1 | 8 | 45 | 4 | 5 |
| Gly α | 1 | 3 | 1 | 16 | 2 |
|  |  |  |  |  |  |
|  |  |  |  |  |  |
| position | 1-^13^C | 2-^13^C | 3-^13^C | 4-^13^C | 5-^13^C |
| Ala CO | 1 | 10 | 56 | 5 | 5 |
| Ala α | 2 | 4 | 1 | 22 | 2 |
| Ala β | 7 | 3 | 2 | 2 | 25 |
|  |  |  |  |  |  |
| Ser CO | 1 | 8 | 48 | 4 | 5 |
| Ser α | 3 | 3 | 2 | 24 | 2 |
| Ser β | 6 | 3 | 1 | 3 | 25 |
|  |  |  |  |  |  |
| Cys CO | 0 | 6 | 33 | 4 | 4 |
| Cys α | 0 | 3 | 1 | 11 | 1 |
| Cys β | 2 | 2 | 1 | 2 | 14 |
|  |  |  |  |  |  |
| Thr CO | 1 | 7 | 13 | 16 | 12 |
| Thr α | 0 | 3 | 1 | 7 | 9 |
| Thr β | 4 | 3 | 2 | 4 | 11 |
| Thr γ2 | 4 | 3 | 3 | 7 | 7 |
|  |  |  |  |  |  |
| Met CO | 0 | 6 | 11 | 13 | 9 |
| Met α | 5 | 2 | -1 | 3 | 4 |
| Met β | 4 | 2 | 1 | 3 | 9 |
| Met γ | 3 | 4 | 2 | 8 | 6 |
| Met ε | 5 | 2 | 1 | 3 | 18 |
|  |  |  |  |  |  |
| Pro CO | 0 | 9 | 8 | 19 | 14 |
| Pro α | 4 | 2 | 1 | 3 | 10 |
| Pro β | 2 | 3 | 1 | 6 | 6 |
| Pro γ | 4 | 2 | 1 | 1 | 10 |
| Pro δ | 2 | 3 | 1 | 13 | 2 |
|  |  |  |  |  |  |
| Asn CO | 0 | 9 | 14 | 20 | 15 |
| Asn α | 2 | 1 | 0 | 5 | 5 |
| Asn β | 5 | 3 | 1 | 5 | 13 |
| Asn γ | 6 | -5 | 5 | 19 | -5 |
|  |  |  |  |  |  |
| Asp CO | 1 | 9 | 17 | 25 | 16 |
| Asp α | 3 | 3 | 0 | 6 | 8 |
| Asp β | 5 | 2 | 1 | 4 | 12 |
| Asp γ | 6 | -5 | 5 | 19 | -5 |
|  |  |  |  |  |  |
|  |  |  |  |  |  |
|  |  |  |  |  |  |
| position | 1-^13^C | 2-^13^C | 3-^13^C | 4-^13^C | 5-^13^C |
| Gln CO | 1 | 8 | 7 | 20 | 14 |
| Gln α | 3 | 3 | 2 | 3 | 9 |
| Gln β | 4 | 3 | 1 | 9 | 7 |
| Gln γ | 5 | 2 | 1 | 2 | 14 |
| Gln δ | 4 | 6 | -4 | 20 | -1 |
|  |  |  |  |  |  |
| Glu CO | 1 | 8 | 6 | 20 | 13 |
| Glu α | 4 | 2 | 0 | 4 | 11 |
| Glu β | 4 | 3 | 1 | 8 | 7 |
| Glu γ | 4 | 2 | 1 | 1 | 12 |
| Glu δ | 4 | 6 | -4 | 20 | -1 |
|  |  |  |  |  |  |
| Lys CO | 1 | 8 | 27 | 9 | 8 |
| Lys α | 3 | 2 | 1 | 9 | 4 |
| Lys β | 4 | 2 | 1 | 2 | 12 |
| Lys γ | 2 | 3 | 3 | 5 | 3 |
| Lys δ | 5 | 2 | 1 | 2 | 13 |
| Lys ε | 3 | 4 | 1 | 11 | 5 |
|  |  |  |  |  |  |
| Arg CO | 0 | 7 | 8 | 13 | 9 |
| Arg α | 4 | 2 | 1 | 3 | 6 |
| Arg β | 2 | 2 | 1 | 3 | 4 |
| Arg γ | 2 | 2 | 2 | 2 | 8 |
| Arg δ | 2 | 4 | 1 | 9 | 2 |
| Arg ζ | 7 | 4 | 12 | 5 | 3 |
|  |  |  |  |  |  |
| Val CO | 1 | 7 | 41 | 4 | 5 |
| Val α | 2 | 4 | 2 | 13 | 3 |
| Val β | 3 | 5 | 1 | 14 | 4 |
| Val γ* | 5 | 2 | 1 | 2 | 17 |
|  |  |  |  |  |  |
| Leu CO | 0 | 9 | 5 | 25 | 4 |
| Leu α | 4 | 2 | 0 | 2 | 7 |
| Leu β | 2 | 5 | 1 | 10 | 2 |
| Leu γ | 1 | 4 | 1 | 8 | 3 |
| Leu δ* | 5 | 2 | 1 | 2 | 15 |
|  |  |  |  |  |  |
|  |  |  |  |  |  |
|  |  |  |  |  |  |
|  |  |  |  |  |  |
|  |  |  |  |  |  |
| position | 1-^13^C | 2-^13^C | 3-^13^C | 4-^13^C | 5-^13^C |
| Ile CO | 1 | 8 | 11 | 15 | 10 |
| Ile α | 3 | 2 | 1 | 3 | 6 |
| Ile β | 2 | 5 | 1 | 15 | 2 |
| Ile γ1 | 3 | 2 | 1 | 2 | 7 |
| Ile γ2 | 6 | 2 | 1 | 2 | 17 |
| Ile δ1 | 2 | 2 | 2 | 4 | 3 |

Values are in %. Errors are estimated to 1 % for ^1^H bound ^13^C, 3 % for others. 1 % for non labeled positions is expected because of natural abundance of ^13^C. Negative values can arise because of noise in the spectra.
